# Challenging the Drug-Likeness Dogma for New Drug Discovery in Tuberculosis

**DOI:** 10.3389/fmicb.2018.01367

**Published:** 2018-07-03

**Authors:** Diana Machado, Miriam Girardini, Miguel Viveiros, Marco Pieroni

**Affiliations:** ^1^Global Health and Tropical Medicine, GHTM, Instituto de Higiene e Medicina Tropical, IHMT, Universidade Nova de Lisboa, UNL, Lisbon, Portugal; ^2^P4T Group, Department of Food and Drug, University of Parma, Parma, Italy

**Keywords:** efflux inhibitors, lipophilicity, medicinal chemistry, proton motive force, rule of five, tuberculosis

## Abstract

The emergence of multi- and extensively drug resistant tuberculosis worldwide poses a great threat to human health and highlight the need to discover and develop new, effective and inexpensive antituberculosis agents. High-throughput screening assays against well-validated drug targets and structure based drug design have been employed to discover new lead compounds. However, the great majority fail to demonstrate any antimycobacterial activity when tested against *Mycobacterium tuberculosis* in whole-cell screening assays. This is mainly due to some of the intrinsic properties of the bacilli, such as the extremely low permeability of its cell wall, slow growth, drug resistance, drug tolerance, and persistence. In this sense, understanding the pathways involved in *M. tuberculosis* drug tolerance, persistence, and pathogenesis, may reveal new approaches for drug development. Moreover, the need for compounds presenting a novel mode of action is of utmost importance due to the emergence of resistance not only to the currently used antituberculosis agents, but also to those in the pipeline. Cheminformatics studies have shown that drugs endowed with antituberculosis activity have the peculiarity of being more lipophilic than many other antibacterials, likely because this leads to improved cell penetration through the extremely waxy mycobacterial cell wall. Moreover, the interaction of the lipophilic moiety with the membrane alters its stability and functional integrity due to the disruption of the proton motive force, resulting in cell death. When a ligand-based medicinal chemistry campaign is ongoing, it is always difficult to predict whether a chemical modification or a functional group would be suitable for improving the activity. Nevertheless, in the “instruction manual” of medicinal chemists, certain functional groups or certain physicochemical characteristics (i.e., high lipophilicity) are considered red flags to look out for in order to safeguard drug-likeness and avoid attritions in the drug discovery process. In this review, we describe how antituberculosis compounds challenge established rules such as the Lipinski's “rule of five” and how medicinal chemistry for antituberculosis compounds must be thought beyond such dogmatic schemes.

## Introduction

Tuberculosis, caused by *Mycobacterium tuberculosis*, remains a major public health problem worldwide. Nowadays, tuberculosis is the leading cause of death due to a single infectious agent. In 2016, the World Health Organization has estimated 10.4 million new cases of tuberculosis with 1.3 million deaths in the same year (WHO, 2017). Moreover, it is estimated that one third of the world's population asymptomatically harbors *M. tuberculosis*, of which 10% will develop active disease in their lifetime. Although drug susceptible tuberculosis can be cured within 6–8 months with the current standard treatment regimen, the emergence of multi- and extensively drug resistant (MDR/XDR) tuberculosis, whose treatment takes at least 20 months with predictable low outcomes (Falzon et al., 2011), poses a great threat to human health and highlights the need to discover and develop new and effective antituberculosis treatments. The underlying reason for the long treatment is the presence of *M. tuberculosis* cells that undergo a reversible metabolic shutdown (Lewis, 2010), resulting in a dormant state. During tuberculosis infection, patients can harbor three different *M. tuberculosis* sub-populations: (i) the first corresponds to the actively growing extracellular bacteria that are usually present within aerated cavities; (ii) the second consists of intermittently growing bacilli; and (iii) the third sub-population corresponds to dormant bacilli that are present in lesions characterized by an acidic environment and under anaerobic conditions, such as in inflammatory lesions or within macrophages (Mitchison, 1979, 1985), and are unaffected by the standard therapy. At this regard, dormancy is different from persistence, as the latter involves a preexisting non-growing subpopulation which displays a non-heritable ability to survive exposure to high concentrations of an antibiotic (Louw et al., 2009). Dormant cells are a double-edged sword: they can remain dormant during the lifetime of an individual, or they can resuscitate at any moment and progress to active tuberculosis. This occurs mainly in immunocompromised patients such as those co-infected with human immunodeficiency virus (HIV), with diabetes, or it can be simply due to aging (Caño-Muñiz et al., 2018). For this reason, the main goal of the ongoing WHO/TB Alliance drug discovery programs is the identification of more-effective drugs with new modes of action with potential to shorten the duration of therapy (Uplekar et al., 2015; Tacconelli et al., 2018) toward the killing of actively growing *M. tuberculosis* and also the effective elimination of the dormant cells. This goes hand in hand with the need to combat the emergence of resistance to new drugs, by identifying gene mutations and molecular drug targets that counteract resistance (Vjecha et al., 2018). Innovative therapies targeting both replicating and dormant *M. tuberculosis* are critical for the development of more effective and shorter treatments, as the most needed basis for future pharmaceutical translation and clinical trials.

Medicinal chemistry remains an important means to achieve better treatments and reach the final goal of tuberculosis eradication. Basic medicinal chemistry rules have been described over many years and used to rationalize the design of many drugs. However, in the case of antituberculosis molecules, consistent adjustments of these rules have been made. Aim of this review is to enrich the tool-kit for antituberculosis drug design, critically analyzing the several structural peculiarities of those molecules in the antituberculosis pipeline, that are seldom found in other therapeutic classes and that make these drugs unique in the medicinal chemistry landscape. Also, a particular focus will be given to the issue of energy depletion, proton motive force (PMF), and transporters in *M. tuberculosis*, as these are hot topics in the current antituberculosis drug discovery.

## State-of-the-art of tuberculosis drug discovery

**Figure 1:**
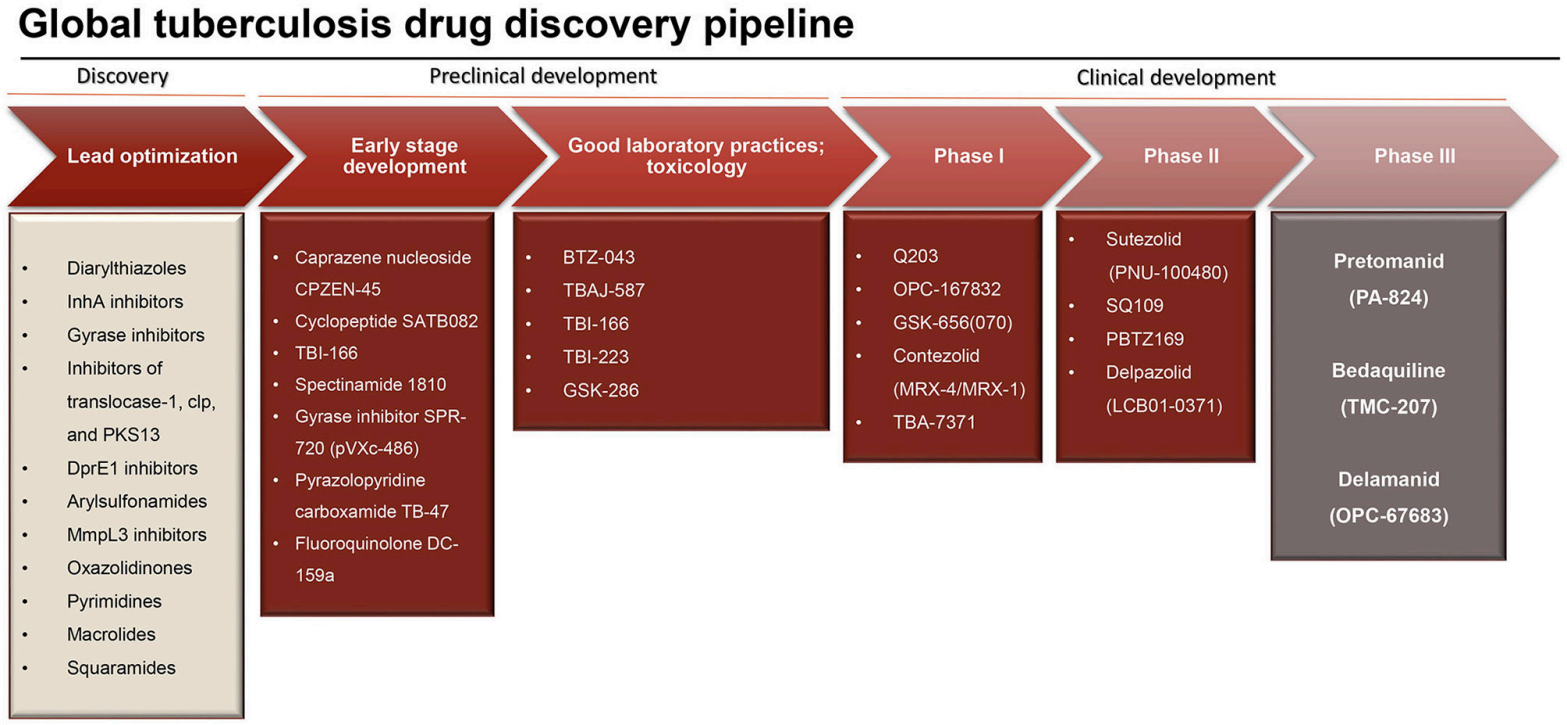
Research and development pipeline for new antituberculosis drugs (adapted from Stop TB partnership, 2014, https://www.newtbdrugs.org/pipeline/clinical).

Tuberculosis drug development has faced a major upsurge in the last two decades resulting in a growing pipeline of new potential antituberculosis drugs (Figure 1) (Laughon and Nacy, 2017). Since the release of *M. tuberculosis* genome sequence (Cole et al., 1998), several efforts have been made for the identification of new key proteins based on gene essentiality. All of a sudden, the high abundancy of drug targets, the majority of which well-validated, gave the feeling that eradication of tuberculosis was just a matter of time. Therefore, massive high-throughput screening campaigns and target-based drug design approaches were employed to discover new lead compounds hitting key enzymes for *M. tuberculosis* survival (Payne et al., 2007; Fischbach and Walsh, 2009). Unfortunately, this approach has not led to any new drug to date, since the great majority of the novel compounds, despite remarkable activity in the biochemical assays, failed miserably to demonstrate the corresponding activity when tested against *M. tuberculosis* in a whole-cell screening assay. One of the reasons for this failure includes the inability to guarantee “druggability” based on the essentiality of the proteins (Keller et al., 2006); undoubtedly, the main reason of this lack of correspondence resides in the extremely challenging task that poses reaching a given target inside the mycobacterial cell. The low permeability of the cell wall appears to be vital for survival of mycobacteria within the host hostile environment, and especially to withstand therapy (Jarlier and Nikaido, 1994). The mycobacterial cell wall has an unique architecture characterized by high content of lipids that work as an impermeable barrier against hydrophilic agents (Jarlier and Nikaido, 1994; Brennan, 2003; Favrot and Ronning, 2012). Along with this basal phenotypic condition, already demanding in terms of targeting, it is known that dormant bacteria adapt their cell wall by accumulating free mycolates and lipoarabinomannan, and transporting them outside the cell (Bacon et al., 2014; Daniel et al., 2014), altering its lipophilic character (Seiler et al., 2003). To make things worse, the cell wall is not the only barrier that a molecule must go through to reach a whatsoever molecular target in the cytoplasm. Indeed, mycobacteria infecting the host reside inside macrophages, and although the macrophage cell membrane is less challenging, its penetration makes the path of an antituberculosis drug bouncier than that of generic antibacterials. Finally, after infection has been established, the formation of granuloma makes the reaching of intracellular targets of extreme complexity. Granulomas are structures which contain the infection but hamper chemotherapeutic action by sequestration of dormant bacilli within the caseous lesions and cavities, where the net of blood vessels is absent (Dartois, 2014). These facts suggest two considerations that are crucial in antituberculosis drug discovery; first, the whole-cell assay fully outclasses target-based methods as the main approach to discover novel antituberculosis drugs. Not by chance, bedaquiline and delamanid, described in details below, were discovered through this method. Second, it appears obvious that lipophilicity represents a very important characteristic to consider in designing new drugs effective against *M. tuberculosis* (Piccaro et al., 2015).

### The phenotypic approach: advantages of the whole-cell screening

**Figure 2:**
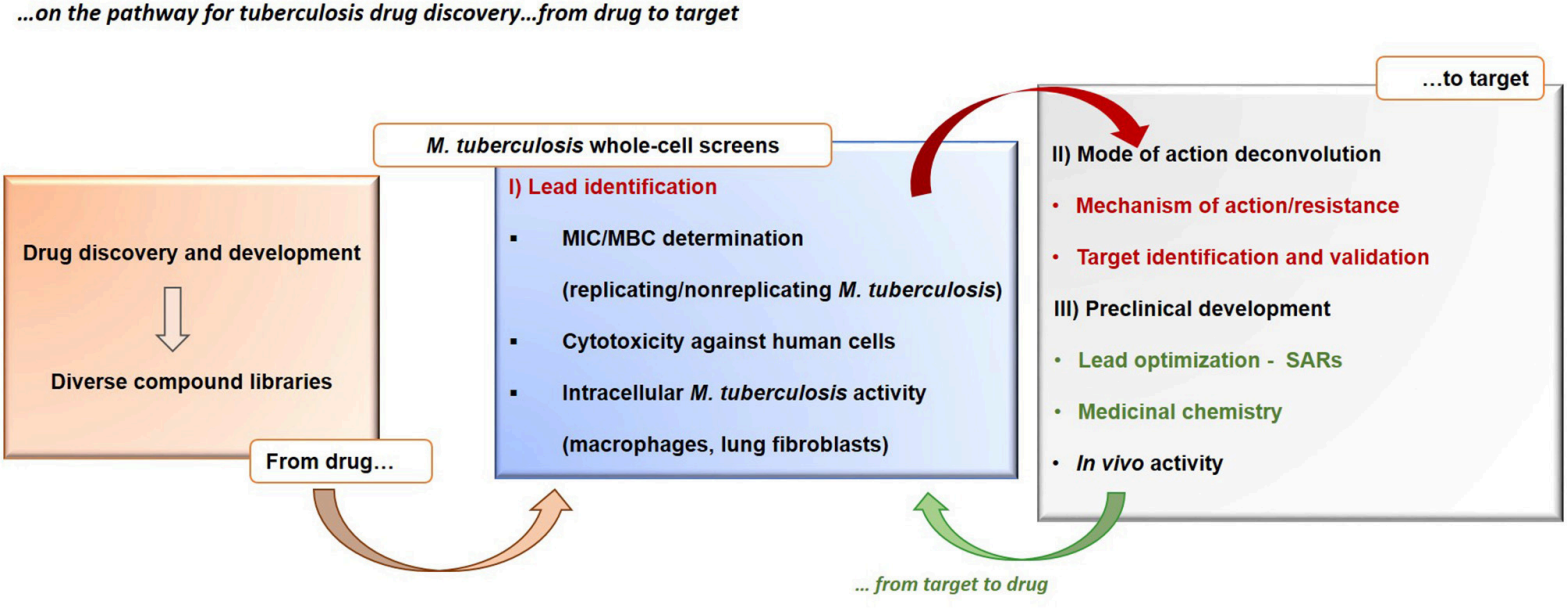
Tuberculosis drug discovery. The figure shows the drug-to-target whole-cell phenotypic approach in the search for new tuberculosis drugs. MBC, minimum bactericidal concentration; MIC, minimum inhibitory concentration. SAR, structure–activity relationship.

Concerning the first aspect, it must be remembered that the majority of antibacterials were discovered through the phenotypic screening of natural extracts, at least at the beginning of the glorious antibiotic era. Further chemical manipulation of these natural compounds has led to the current antibacterial arsenal. This strategy has been demonstrated to be much more successful especially in the case of tuberculosis (Payne et al., 2007; Koul et al., 2011), as evidenced by the six new drugs in phase I, II or III of clinical trials, specifically developed for the treatment MDR tuberculosis, all discovered starting by the screening of their whole-cell activity (Laughon and Nacy, 2017). The main reason for this success is that, instead of using the overly reductionist approach of finding a drug that hits a single target, screening directly for whole-cell activity allow to find compounds with pleomorphic mechanisms of action hitting multiple targets in different pathways to achieve the desired outcome and bypasses the general problems associated with drug failure, such as low permeability, drug efflux, or bacterial metabolic plasticity when targeting its central metabolism (Mukherjee et al., 2016). The phenotypic approaches in tuberculosis drug discovery rely on two main steps, that come before the actual clinical phases (Figure 2): (i) the testing of diverse chemical libraries of compounds, using cell-based screens, in order to determinate the minimum inhibitory concentrations (MICs), followed by (ii) elucidation of the compound mode of action, validation of its molecular target and identification of their mode of resistance. The determination of the molecular target(s) of the candidate molecules is a main challenge of the phenotypic whole-cell screening and is instrumental for further optimization. At this end, new technologies, including *in vitro* resistance mutation analysis (Andries et al., 2005; Manjunatha et al., 2006), knockout studies and construction of conditional mutants (Singh and Mizrahi, 2017), cytological profiling (Nonejuie et al., 2013), analysis of transcriptional (Boshoff et al., 2004; Koul et al., 2014), and proteome responses (Koul et al., 2014) are proving successful. Usually these demanding approaches rely on the generation of resistant mutants by exposing *M. tuberculosis* to high concentrations of the compound and the identification of resistance-associated mutations by whole genome sequencing (O'Malley and Melief, 2015). However, it has been observed that for some hit compounds is not possible to generate resistant *M. tuberculosis* mutants, making the target identification and validation problematic. In these cases, post-genomic tools have the potential to aid in the identification of the target(s) and compound mode of action, e.g., using transcriptional profiling (Boshoff et al., 2004) or high-throughput metabolomic analysis (Zampieri et al., 2018), before and after drug exposure, and reveal *M. tuberculosis* transcriptional profiles or metabolic responses allowing to correlate with the compound mechanism of action. Another challenge of the phenotypic screenings is to identify the right *in vitro* conditions that are relevant *in vivo*, e.g., compounds that target metabolic enzymes may require specific growth conditions (Pethe et al., 2010). Moreover, the phenotypic screening of compounds has the potential to deliver thousands of new hits, however, many of these may have cytotoxic effects, that can be identified using counter-screening methods (e.g., screening for toxicity against eukaryotic cell lines, effect on membrane permeability, and red blood cell hemolysis assays) to achieve good selectivity and specificity (Hurdle et al., 2011; Koul et al., 2014).

### Lipophilicity and *M. tuberculosis*: the odd couple

Lipophilicity, although regarded as an ostracized characteristic in medicinal chemistry, plays a pivotal role in the design of novel antituberculosis compounds. Overall, it can be stated that the lipophilicity of a molecule corresponds the partitioning into *M. tuberculosis* cell wall and, possibly, into the hydrophobic phases of *caseum*, and suggests that general lipophilic character should be pursued in drugs for antituberculosis treatment. This notion may sound odd, especially if one considers the first-line antituberculosis drugs: isoniazid and pyrazinamide both have negative ClogP, whereas ClogP of ethambutol is 0.35. However, it has been deeply demonstrated that compounds with antituberculosis activity are more lipophilic than the inactive ones (Ekins et al., 2011), and not only in general, but also within a drug class, lipophilic derivatives are in general more active against mycobacteria than their more hydrophilic counterparts (Mao et al., 2009; Lilienkampf et al., 2010, 2012; Pieroni et al., 2010, 2011, 2017). Below, a critical analysis of the current hot biology challenges (energy depletion, PMF, and drug transporters) and their interconnection with the lipophilicity of molecules is reported.

## *M. tuberculosis* biology challenges: focus on energy depletion

### Energy metabolism as a new drug-target pathway in tuberculosis drug discovery

**Figure 3:**
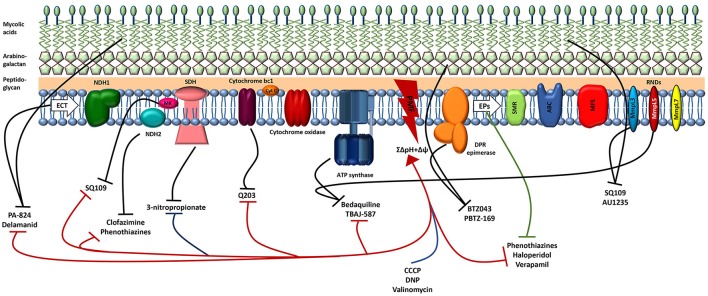
Schematic illustration of the *M.tuberculosis* cell membrane, including the electron transport chain (ETC), efflux pumps (EPs), and the site of action of several antituberculosis drugs. The great majority of the drugs (approved for tuberculosis, new or repurposed) target both enzymes (black lines) and the PMF (red). Blue line shows the classic protonophores disrupting the PMF and the green line indicates the efflux inhibitors that target several mycobacterial efflux pumps. By damaging the cell membrane, the lipophilic drugs will affect the activity of several membrane enzymes such as those involved in the ETC and efflux pumps responsible for the extrusion of several compounds from the cell. The inhibition of any component of the ETC reduces energy production and disrupts membrane potential. Consequently, the disruption of the PMF reduces the activity of the efflux pumps. Regarding the mode of action of the compounds see the text for details. NDH1, NADH dehydrogenase type I; NDH2, NADH dehydrogenase type II; SDH, succinate dehydrogenase; MK, menaquinone; Cyt C, cytochrome c; PMF, proton motive force; DPR, decaprenylphosphoryl-β-d-ribose 2′-epimerase; SMR, small multidrug resistance; ABC, ATP binding cassette; MFS, major facilitator superfamily; RND, resistance-nodulation and cell division.

Recent advances have populated the tuberculosis drug discovery pipeline with promising drug candidates and new interesting target/s or pathways. Among these, the complex and waxy cell wall of *M. tuberculosis* has emerged as an intriguing source of new drug targets (Table 1). Several drugs whose mechanism of action is known to affect the mycobacterial cell membrane and metabolic energy and respiration are now in the pipeline, highlighting a major role of energy metabolism as a new drug target pathway in mycobacteria (Figure 3).

**Table 1:** Main anti-tuberculosis drug candidates in clinical development reported in the review, their mechanism of action, molecular targets, and mode of resistance.

**Compound**	**Class**	**Identification strategy**	**Mode(s) of action**	**Target(s)**	**Mechanism of resistance**	**References**
Bedaquiline	Diarylquinoline	Whole-cell screening of prototypes of different chemical series	(i) Inhibition of ATP biosynthesis (ii) Efflux inhibition	Subunit *c* of the ATPase	(i) Mutations in the subunit *c* of the ATPase (ii) Mutations in the transcriptional repressor Rv0678 (MmpR5) (iii) Substrate of efflux pumps—MmpS5-MmpL5 system	(Andries et al., 2005) (Andries et al., 2014) (Hartkoorn et al., 2014)
TBAJ-587	Diarylquinoline	Optimization of diarylquinolines	Inhibition of ATP biosynthesis	Subunit *c* of the ATPase	(i) Mutations in the subunit *c* of the ATPase (ii) Mutations in the transcriptional repressor Rv0678 (MmpR5) (iii) Substrate of efflux pumps—MmpS5-MmpL5 system	(Tong et al., 2017) (Choi et al., 2017) (Sutherland et al., 2018)
PA-824	Nitroimidazole	Whole-cell screening of a series of 3-substituted nitroimidazopyrans	(i) Pro-drug (ii) Inhibition of ATP biosynthesis (iii) Respiratory poison by nitric oxide (NO) production	Deazaflavin (cofactor F(420 dependent nitroreductase (Ddn)	(i) Mutations in the activator *ddn* (ii) Mutations in the genes *fgd1, fbiA, fbiB*, and *fbiC* encoding the 6-phosphate dehydrogenase Fgd1 required for cofactor F420 biosynthesis	(Stover et al., 2000); (Manjunatha et al., 2006); (Manjunatha et al., 2009), (Singh et al., 2008) (Choi et al., 2001) (Choi et al., 2002)
OPC-67683	Nitroimidazole	Whole-cell screening for inhibitors of mycolic acid biosynthesis	(i) Pro-drug (ii) Inhibition of ATP biosynthesis (iii) Respiratory poison by NO production	Deazaflavin (cofactor F(420)) dependent nitroreductase (Ddn)	(i) Mutations in the activator *ddn* (ii) Mutations in the genes *fgd1, fbiA, fbiB*, and *fbiC* encoding the 6-phosphate dehydrogenase Fgd1 required for cofactor F420 biosynthesis	(Matsumoto et al., 2006) (Stover et al., 2000); (Singh et al., 2008) (Choi et al., 2001) (Choi et al., 2002)
Q-203	Imidazopyridine amide	Phenotypic screening of various commercial chemical libraries in infected macrophages	Inhibition of the cytochrome *bc*1 complex (complex III)	Cytochrome *b* subunit (QcrB) of the cytochrome *bc*1 complex	(i) Mutations in the *qcrB* gene (ii) Substrate of efflux pumps	(Pethe et al., 2013) (Jang et al., 2017)
SQ-109	1,2-ethylene diamine	Whole-cell screening of ethambutol derivatives	(i) Inhibits mycolic acid biosynthesis (ii) Inhibition of menaquinone synthesis	(i) Mycobacterial trehalose monomycolate transporter MmpL3 (ii) MenA and MenG	(i) Mutations in the *mmpL3* gene (ii) Mutations in *menA* and *menG* genes	(Protopopova et al., 2005) (Sacksteder et al., 2012) (Li K. et al., 2014) (Li W. et al., 2014)
BTZ-043	Benzothiazinone	Whole-cell screening of a series of sulfur-containing heterocycles	(i) Pro-drug (ii) Inhibit arabinan biosynthesis	Decaprenylphosphoryl-β-d-ribose 2′-epimerase (DprE1)	Mutations in the activator encoding the gene *dprE1*	(Makarov et al., 2009) (Trefzer et al., 2010)
PBTZ-169	Benzothiazinone	Whole-cell screening of a new generation of BTZ derivatives from BTZ043	Inhibit arabinan biosynthesis	Decaprenylphosphoryl-β-d-ribose 2′-epimerase (DprE1)	Mutations in the activator encoding gene *dprE1*	(Makarov et al., 2014)
AU-1235	Adamantyl urea	Whole-cell screening of a diverse commercial compound libraries	Inhibits mycolic acid transport	Mycobacterial trehalose monomycolate transporter MmpL3	Mutations in the *mmpL3* gene	(Grzegorzewicz et al., 2012)
TB-47	Pyrazolopyrimidine	Phenotypic screening	–	–	–	(Tang et al., 2015) (Zhang et al., 2017)

### Collapsing *M. tuberculosis* proton motive force

The development of more efficient and shorter treatments for tuberculosis requires the rapid killing of actively growing *M. tuberculosis* and the effective elimination of persistent dormant cells. Ideally, a new antituberculosis drug needs to be active against both replicating and non replicating *M. tuberculosis*, penetrate within tissues and granulomas, and show a low and slow propensity for drug resistance (de Carvalho et al., 2011; Feng et al., 2015; Moreira et al., 2016; Mukherjee et al., 2016). The shift to a dormant state involves several phenotypic changes that reduce bacterial metabolic activity and modify the overall architecture of the cell wall. In this sense, the cellular targets of the current antituberculosis drugs that are required for *M. tuberculosis* growth and survival during active infection are not essential for the survival of dormant cells, thus rendering the bacteria phenotypically drug tolerant (Gengenbacher and Kaufmann, 2012). Nevertheless, the maintenance of bacterial membrane integrity and homeostasis is essential regardless the metabolic status of the cell (Hurdle et al., 2011). The recognition of some drugs targeting the bacterial cell membrane (e.g., daptomycin, televancin, bedaquiline, clofazimine) validates the membrane as an antibacterial target (Yawalkar and Vischer, 1979; Andries et al., 2005; Hawkey, 2008; Zhanel et al., 2010) and a number of antituberculosis drugs in the pipeline target membrane proteins (Lechartier et al., 2014). The mycobacterial cell wall is rich in surface lipids, long chains of mycolic acids, and peptidoglycan, thus is not surprising that they display a preference for lipophilic molecules leaving behind the more hydrophilic ones (Jarlier and Nikaido, 1994; Brennan, 2003). The PMF is established through the development of the transmembrane proton gradient which occurs due to the movement of electrons through the electron transport chain, resulting in the formation of the membrane potential (Mitchell, 1967). Oxidative phosphorylation is the main source of energy production in mycobacteria. *M. tuberculosis* is an obligate aerobic pathogen and consequently it depends on oxidative phosphorylation for growth and survival. During oxidative phosphorylation, the electrons derived from NADH are fed into the electron transport chain by the type II NADH dehydrogenase (NDH-2), leading to the reduction of the menaquinone pool (MK/MKH). Additionally, the MK/MKH can also be reduced by alternative electron donors, e.g., via the succinate dehydrogenase (SDH). Electrons can be transferred directly from the MK/MKH to the cytochrome *bc*1-*aa*3 complex or alternatively, the oxygen can be reduced by a cytochrome *bd*-type terminal oxidase, which directly accepts electrons from the MK/MKH (Black et al., 2014; Bald et al., 2017; Iqbal et al., 2018). The proton gradient generated through oxidative phosphorylation leads to ATP synthesis via the ATP synthase which is responsible for the conversion of the electrochemical potential energy generated by the PMF into chemical energy in the form of ATP (Feniouk et al., 2007). The PMF is the sum of two gradients: an electrical potential (Δψ) and a transmembrane proton gradient (ΔpH). The majority of bacteria is able to maintain a relatively neutral intracellular pH that is controlled by the activity of ion transport systems which facilitate the entry or exit of protons (Booth, 1985). At neutral pH, the PMF is predominantly in the form of membrane potential, but as the external pH drops, the transmembrane proton gradient increases and the membrane potential decreases to maintain a constant PMF and vice-versa (Bakker and Mangerich, 1981). Under normal growth conditions and at neutral pH mycobacteria generates a PMF of ~-180 mV (Rao et al., 2001). Under hypoxia*, M. tuberculosis* generates a total PMF of −113 mV. Dissipation of the PMF leads to a rapid loss of cell viability and cell death. Thus, energy metabolism and ATP production through the PMF, which is established by the electron transport chain, significantly contribute to drug susceptibility in *M. tuberculosis* (Black et al., 2014). In this sense, combinations of dissipaters of membrane potential with dissipaters of the transmembrane gradient might be highly synergistic (Farha et al., 2013) against *M. tuberculosis* infections. This multiple targeting is of utmost importance in overcoming the development of drug resistance because the main target is not an enzyme *per se*, but rather the product of complex biosynthetic pathways (Feng et al., 2015; Moreira et al., 2016; Mukherjee et al., 2016). Mycobacteria are highly sensitive to compounds that dissipate the membrane potential as the uncouplers/protonophores, in addition to the binding to their enzyme targets (de Carvalho et al., 2011; Rao et al., 2013; Feng et al., 2015; Moreira et al., 2016; Mukherjee et al., 2016). Feng et al. (2015) have demonstrated that the antituberculosis activity of uncouplers/protonophores, compounds that target the PMF, is directly proportional to their lipophilicity. Thioridazine, a compound that target the NDH-2, causes dissipation of the membrane potential and cell death, suggesting NADH as an important electron donor for the generation of the membrane potential during hypoxia (Rao et al., 2013). Further, inhibitors of the SDH, as 3-nitropropionate, are also able to dissipate the membrane potential under hypoxia, also suggesting SDH as another important generator of the membrane potential in nonreplicating conditions (Eoh and Rhee, 2013; Pecsi et al., 2014). Other examples of uncouplers are bedaquiline and clofazimine, another NDH-2 inhibitor (Feng et al., 2015). Uncouplers/protonophores are highly bactericidal toward replicating and nonreplicating (active and dormant) *M. tuberculosis* cells, further highlighting the importance of the membrane potential in mycobacterial viability. Although it is generally expected that this type of compounds show toxicity toward human cells, several of the US Food and Drug Administration-approved drugs act as uncouplers in addition to the inhibition of targeting enzymes (Feng et al., 2015).

## Targeting mycobacterial efflux pumps

Compounds that inhibit bacterial energy metabolism could be highly synergistic in combination with the current treatment regimens due to their interference with efflux of drugs. The concept of enhancing the activity of the current antituberculosis drugs by employing efflux inhibitors is quite appealing for several reasons. As for many bacterial species, the main mechanisms associated with drug resistance in *M. tuberculosis* involves the development of mutations in target genes (Böttger, 2011) and the activity of efflux transporters capable of pumping antibiotics out of the cell (Louw et al., 2009; Viveiros et al., 2012; Costa et al., 2016). Efflux pumps are now increasingly recognized as playing a significant role in the resistance levels of *M. tuberculosis* to antibiotics (Machado et al., 2017, 2018), being associated with the emergence of multidrug resistance phenotypes (Viveiros et al., 2002; Machado et al., 2012). It was shown that the decrease of intracellular concentrations of antituberculosis drugs due to an adaptive increase of efflux activity allows the bacteria to survive in the host for a longer period of time under antibiotic pressure (Machado et al., 2012), increasing the probability of selecting spontaneous mutants with high-level resistance. Also, since they do not kill bacteria, these agents are likely exempt from the raise of resistance (Zumla et al., 2016). Efflux pumps are energized by the hydrolysis of ATP or by the PMF, therefore drugs that are able to disrupt the PMF or block the production of ATP exhausting energy supply can be an effective strategy to evade drug efflux (Black et al., 2014; Machado et al., 2016; Pule et al., 2016). Energy in the form of ATP is used by the primary transporters (ATP binding cassette efflux transporter); on the contrary, the secondary transporters, e.g., the resistance nodulation cell division and the major facilitator superfamily of efflux transporters, act based on the electrochemical gradient generated by the PMF (Kumar and Schweizer, 2005; Piddock, 2006). Therefore, compounds inhibiting oxidative phosphorylation may indirectly interfere with efflux activity. *M. tuberculosis* drug resistance can be reduced in the presence of efflux inhibitors such as thioridazine, chlorpromazine, flupenthixol, and haloperidol (antipsychotic drugs), verapamil (an antiarrhythmic drug) (Machado et al., 2016), and the typical protonophores carbonyl cyanide *m*-chlorophenyl hydrazone (CCCP), 2,4-Dinitrophenol (DNP), and valinomycin (Pule et al., 2016). Phenothiazines are calcium channel blockers and inhibit efflux activity by reducing the transmembrane potential. Verapamil is an inhibitor of the ABC transporter Pgp in mammalian cells (Endicott and Ling, 1989). It has been shown to be the most potent mycobacterial inhibitor of efflux, being able to enhance the inhibitory activity of isoniazid (Machado et al., 2012, 2016, 2018), rifampicin (Louw et al., 2011; Machado et al., 2016, 2018) and the novel drug bedaquiline (Gupta et al., 2013) by several folds. It has also been shown that the addition of verapamil accelerates the bactericidal and sterilizing activities of tuberculosis therapy in the mouse model (Gupta et al., 2013). Using docking studies, it found that verapamil can bind to the *M. tuberculosis* Rv1258c efflux transporter (Singh et al., 2014). Very recently it was shown that verapamil disrupts *M. tuberculosis* membrane potential (Chen et al., 2018). The phenothiazines inhibit the NDH-2 enzyme (Weinstein et al., 2005; Warman et al., 2013) causing dissipation of the membrane potential. Thioridazine and chlorpromazine have been shown to have active efflux inhibition properties and to inhibit the *in vitro* growth of *M. tuberculosis* strains alone or in combination with anti-mycobacterial drugs (Amaral et al., 2007; Machado et al., 2016). Thioridazine demonstrates significant activity against multidrug resistant tuberculosis in a murine model (van Soolingen et al., 2010) and, in addition, it was shown that these compounds were able to increase the intracellular levels of ethidium bromide in *M. tuberculosis* (Machado et al., 2016, 2018). The inhibition of efflux and its antituberculosis activity were correlated with ATP depletion providing a link between the inhibition of efflux activity and the interference with membrane energetics (Machado et al., 2016). Likewise, Lu et al. have demonstrated the same effect for the ATP synthase inhibitor bedaquiline in *M. smegmatis* (Lu et al., 2014). Efflux activity also mediates the resistance of intracellular *M. tuberculosis* to a variety of antituberculosis drugs, including isoniazid, rifampicin, moxifloxacin, PA-824, linezolid, and bedaquiline. Adams et al. has demonstrated that the selective pressure exerted by the macrophage on internalized *M. tuberculosis* can induce the bacteria efflux pumps and thus drug tolerance. The addition of the efflux inhibitor verapamil or its metabolites was able to reverse the tolerance to isoniazid and rifampicin (Adams et al., 2011, 2014) highlighting the importance and benefits of combined therapeutic regimens (Martins et al., 2008). The delivery of antituberculosis drugs in conjunction with efflux inhibitors may provide an effective therapeutic approach that obviates the serious side effects resulting from the current chemotherapeutic applications and provides a new approach for the therapy of multidrug resistant infections. These compounds are ideal candidates that can be tested in combination with conventional antibiotics not only because they promote the retention of the co-administered antibiotics but also because these compounds enhance the killing activity of the macrophages. Disruption of the PMF with efflux inhibitors, due to inhibition of the respiratory chain, results in the inhibition of energy-dependent efflux systems in *M. tuberculosis*, therefore the retention of antibiotics subject to active efflux is a suitable option for drug combination regimens needed to fight MDR *M. tuberculosis*. This will prevent emergence of acquired drug resistance and ultimately eliminate *M. tuberculosis* once used in combination with the antituberculosis drugs. The design of innovative therapies based on the use of efflux inhibitors targeting both replicating and dormant *M. tuberculosis*, exploring their dual activity—PMF dissipation and efflux inhibition—will be a valuable tool for the development of more effective and shorter treatments for tuberculosis and to fight multi- and extensively drug resistant tuberculosis.

## Medicinal chemistry for antituberculosis drugs: mind the fat

Cheminformatics studies have shown that drugs endowed with antituberculosis activity have the peculiarity of being more lipophilic than many other antibacterial compounds (Ekins et al., 2011; Goldman, 2013; Lakshminarayana et al., 2015; Piccaro et al., 2015). The improved antituberculosis activity of lipophilic compounds is linked to improved cell penetration into the extremely waxy mycobacterial cell wall. As previously seen, in some cases the interaction of the lipophilic moieties with the membrane alters its stability and functional integrity due to the disruption of the PMF, resulting in cell death (Hurdle et al., 2011). Lipophilicity has been often considered the Achilles' heel of every drug class. Over the years, “empirical rules” have been established regarding the acceptable physiochemical properties and structural motifs for drugs, to better understand and manage drug development risks associated with their molecular properties (Hansch et al., 1987). These efforts have led to the release of the almighty Lipinski's “rule of five” (Lipinski et al., 1997), that gives a rough evaluation of the potential of a small molecule to be absorbed after oral administration. According to the “rule of five,” absorption and/or permeation are more likely if a compound complies with one or more of the following properties: molecular weight (MW) < 500 Da, ClogP < 5, hydrogen bond donors (HBD) < 5, or hydrogen bond acceptors (HBA) < 10. After this first attempt to rationalize the absorption as a function of the molecular properties, implementations were made. Veber's rule added that compounds with < 10 rotatable bonds and a polar surface area (PSA) < 140 Å^2^ are more likely to be orally bioavailable (Veber et al., 2002). Gleeson revised some of these parameters, suggesting an improvement of the ADMET characteristics when MW < 400 and CLogP < 4 (Gleeson, 2008). Since its enunciation, the Lipinski's “rule of five” has been considered a standard rule of thumb to rapidly assess whether a molecule has a good balance of solubility and permeability, and has driven the rational design of novel chemical entities endowed with biological activity. However, adherence to the “rule of five” is only one of the characteristics embraced by the notion of drug-likeness. Indeed, other structural red flags, such as the presence of functional groups known to be metabolically reactive, or bulky, or toxic *per se*, must be considered when the synthesis of a drug-like molecule is pursued. Violation(s) of the above described parameters are considered detrimental in view of further development, and are used to set up *in silico* filtering of large libraries of compounds by many pharmaceutical companies. If the “rule of five” is one of the most significant statistical means for the design of new drugs, a common complain, sometimes used to justify the synthesis of poorly drug-like molecules, is that the “rule of five” does not apply to antibiotics or, more in general, to antibacterials. Although mostly true, this objection is incomplete and it should be rephrased: to be more accurate, the “rule of five” do not apply to natural compounds, and since the majority of antibiotics are natural compounds or thereof derivatives, then this rule does not apply to antibacterials. On the contrary, molecules coming from the synthesis are strongly suggested to follow the “rule of five,” in order to prevent attritions in the pharmaceutical development (i.e., among antibacterials, quinolones, and oxazolidindiones). Despite these facts, a close analysis of the currently marketed drugs used for the treatment of several diseases, that must be by definition harmless and effective, shows that a small percentage of them present a panoply of “forbidden” functionalities (structural alerts) and “unacceptable” properties, including endoperoxides, compounds containing nitro- or isothiourea-moieties, large macrocyclic ring systems and high lipophilicity that fall beyond the drug-likeness dogma (Doak et al., 2014; Hoagland et al., 2016). Of this small percentage, a class of molecules for which the “rule of five” and other basic drug design rules are largely amended is that of antituberculars. Below we introduce a series of molecules that will be critically evaluated for their adherence to the “rule of five” and their drug-likeness. Some of them are advancing in the clinical trials and likely will succeed in the clinic, whereas others are still in the preclinical studies or in the hit-to-lead optimization process. Those chemical entities originating from old antibacterial classes (i.e., novel quinolones or oxazolidindiones) will not be taken into consideration in this critical review, whereas the chemical characteristics of some molecules to be potentially used as adjuvants in antituberculosis treatment will be analyzed as well.

### Antituberculosis drugs: a class of its own

#### Bedaquiline and TBJ-587

**Figure 4:**
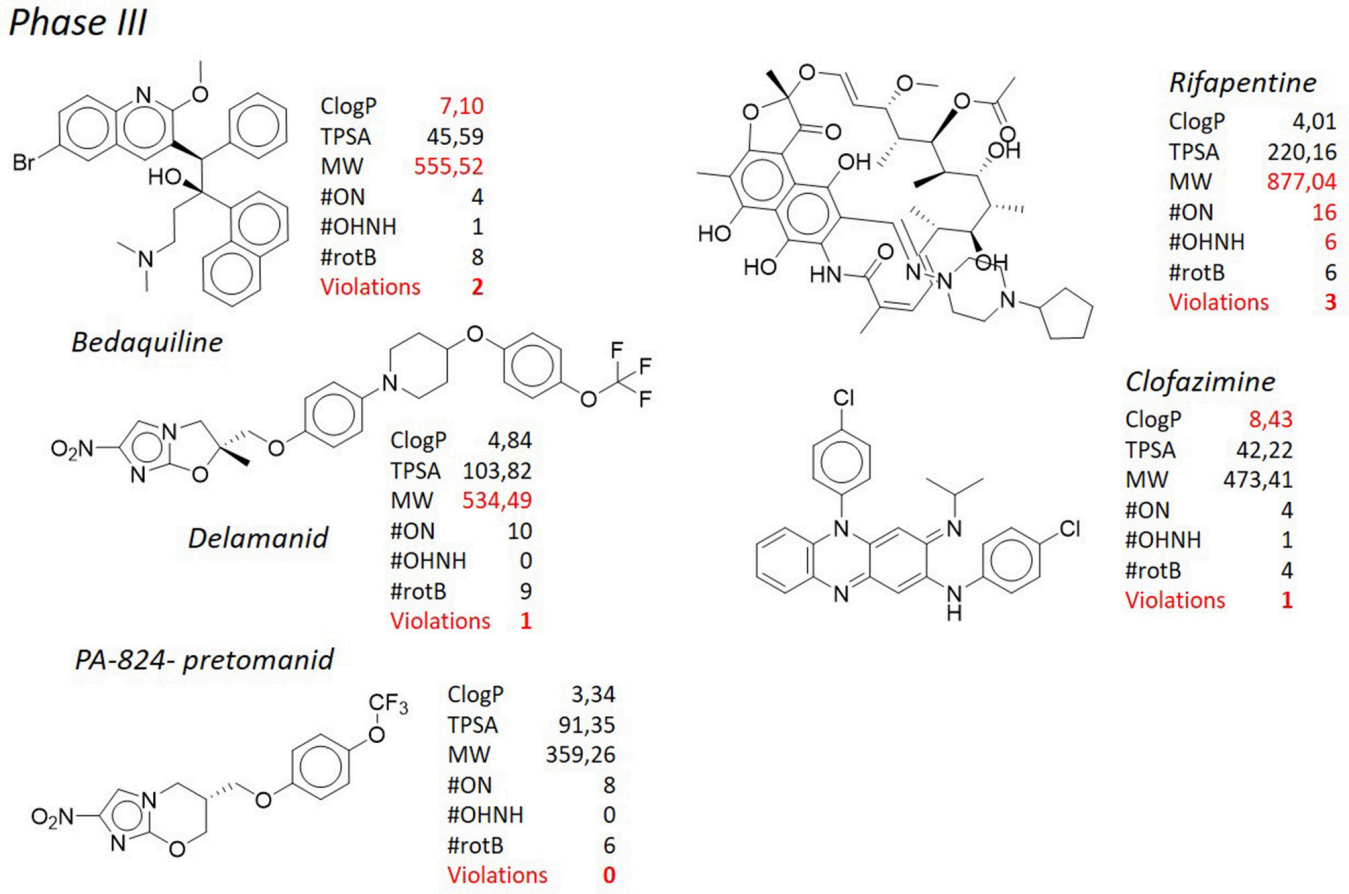
TB pipeline: molecules in phase III clinical trials. Source: https://www.newtbdrugs.org/pipeline/clinical. For each molecule physicochemical characteristics are reported, along with the violation to the “rule of five.” Physicochemical properties calculated at http://www.molinspiration.com/cgi-bin/properties.

Since the introduction of rifampicin in 1967 (Sensi, 1983), bedaquiline (Sirturo®), belonging to the class of the diarylquinolines (DAQ) is the only new chemical entity (NCE) developed for the treatment of tuberculosis that has reached the market (Mahajan, 2013), although its use is restricted to the treatment of multi- and extensively drug resistant tuberculosis (Figure 4). Unfortunately, after its introduction in the clinical practice, an unexpected number of abnormal deaths were reported, probably due to the serious side effects associated with significant cardiac arrhythmia (Fox and Menzies, 2013; Kakkar and Dahiya, 2014; Guglielmetti et al., 2017). The diarylquinolines were identified in a phenotypic screening of various compounds for potential antituberculosis activity (Andries et al., 2005) and the lead compound bedaquiline (also called TMC207, R207910, or compound J), was developed by Janssen Infectious Diseases and the TB Alliance. Bedaquiline is an ATP synthase inhibitor and binds to subunit c (AtpE) of the mycobacterial ATP synthase enzyme (complex V) thus blocking its action (de Jonge et al., 2007; Koul et al., 2007). Bedaquiline has an MICs of 0.03–0.12 μg/ml against drug-sensitive and drug-resistant *M. tuberculosis* strains, both replicating and nonreplicating (Koul et al., 2008), and it shows an exceptional activity *in vivo*. During hypoxia, diarylquinolines inhibit ATP synthesis but even at high concentrations they had no significant effect on membrane potential (Koul et al., 2008; Hards et al., 2015). Recently, it was demonstrated that upon bedaquiline exposure, the mycobacteria tend to minimize the consumption of cellular ATP and at same time enhance the capacity of ATP-generating pathways, which contributes to maintain bacterial viability in spite of antibiotic stress (Koul et al., 2014). It was also showed that mycobacteria grown on lipid-rich media display enhanced bedaquiline-mediated killing indicating a role of energy source on mycobacterial susceptibility. This type of compounds inhibits the ATP synthase not only in bacteria but also in mitochondria (Matsuno-Yagi and Hatefi, 1993), however, it was shown (Haagsma et al., 2009) that bedaquiline may not elicit ATP synthesis-related toxicity in mammalian cells. Mycobacteria that are resistant to bedaquiline *in vitro* have mutations in the *atpE* gene, which encodes the subunit c of ATP synthase (Petrella et al., 2006; Huitric et al., 2010). However, resistant strains with no detectable mutations in the complete F_0_ ATP synthase operon (*atpB, atpE*, and *atpF* genes) and the F_1_ ATP synthase operon (*atpH, atpA, atpG, atpD*, and *atpC* genes) were already detected. Recently, clofazimine-resistant *M. tuberculosis* mutants isolated *in vitro*, were found to be also resistant to bedaquiline. Mutations in the transcriptional regulator Rv0678 (MmpR5), with concomitant upregulation of the efflux pump MmpL5 accounted for this cross-resistance (Andries et al., 2014; Hartkoorn et al., 2014). Bedaquiline is a pure enantiomer with two chiral centers, with a functionalized lateral chain containing a tertiary amine. Three cycloaromatic rings (a naphthyl, a quinolone, and a phenyl ring) represent the bulky core of the molecule, and, in addition, a bromine atom is attached at the C6 of the quinolone ring, leading to a remarkably high degree of lipophilicity (ClogP = 7.10). Of note, the high ClogP is obtained despite the fact that hydrophilic functional groups such as a hydroxyl moiety and a tertiary amino group are present in the molecule. In addition to the high ClogP, the molecular weight (MW = 555.52) accounts for the second violation of the Lipinski's “rule of five.” Nevertheless, this molecule has reached the market and nowadays is used as last resort therapy in co-administration with first line drugs for the treatment of multidrug resistant tuberculosis. The presence of the bromine atom is another peculiarity that makes this molecule a “structure of its own.” Bromine is not considered a “privileged” moiety in medicinal chemistry, since its bulky and hydrophobic nature. Heavy halogens like bromine and iodine in drugs have been associated with phototoxicity, photoreactivity, reactive metabolite formation, positivity to Ames test, and with general safety issues (Pal et al., 1996). Side effects seem to be associated with the extremely long half-life and low water solubility (precipitation in the lungs resulting in pulmonary toxicity) of these molecules, due to the high ClogP of bromine/iodine, rather than to their chemical nature. In addition, during metabolism, the bromine atom may be displaced very easily by a reactive intermediate such as quinone methide. Likely for these reasons there are only 27 approved drugs containing this moiety (source https://www.drugbank.ca/), that is frequently replaced by “safer” halogens such as chlorine and fluorine. Of note, 4 of these bromine-containing molecules were withdrawn from the market due to toxicity. From the very preliminary SAR of these DARQs, any attempt to replace the bromine atom was detrimental. Indeed, substitution with more polar moieties, such as the OCH_3_, led to a >100-fold raise of the MIC (MIC of the 6-OCH_3_ analog = 20.17 μg/mL). Also the substitution with a chlorine, which shares a similar stereo-electronic nature, led to a sharp decrease in the potency (MIC of the 6-Cl analog = 10.51 μg/mL). The same poor results are obtained when the naphthyl ring is adorned with hydrophilic moieties or replaced by less lipophilic aromatic ring. All of these findings corroborate the idea that, along with the suitable interaction of the molecule with its molecular target, lipophilicity is a main feature ensuring antituberculosis potency. Besides bedaquiline, another DARQ, namely TBAJ-587 (**Figure 8**, Choi et al., 2017; Tong et al., 2017; Sutherland et al., 2018) is currently under pre-clinical investigation by Janssen Pharmaceuticals and TB Alliance. This next-generation DARQ was designed and synthesized in order to improve the drug-likeness of bedaquiline, although maintaining its remarkable antituberculosis activity. The main features characterizing the structure of bedaquiline were maintained, however replacement of the naphthyl ring with a dimethoxy pyridine allowed to obtain a molecule with a lower ClogP (5.21) and, therefore, with improved pharmacokinetic characteristics. Nevertheless, as for the parent molecule, still two violations of the “rule of five” (ClogP and MW) are present.

#### Delamanid, PA-824, Q-203, and TB-47

Shortly after bedaquiline, delamanid (Figure 4, Matsumoto et al., 2006; Gler et al., 2012; Skripconoka et al., 2013; deltyba®, former OPC-67683) has received conditional approval for the treatment of multi- and extensively drug resistant tuberculosis by the European Medicines Agency (Sotgiu et al., 2015). Also in this case, severe side effects such as cardiac arrhythmia and general central nervous system toxicity, especially when used in combination with isoniazid or fluoroquinolones (Harausz et al., 2015), have cooled down the initial enthusiasm raised by the introduction in therapy of this novel antituberculosis agent. In addition, mutations in the *M. tuberculosis* genome causing resistance to delamanid have been recently documented (Bloemberg et al., 2015). Delamanid, developed by Otsuka Pharmaceutical Co., and its precursor PA-824 (pretomanid, Figure 4), developed by PathoGenesis Co (currently Novartis AG), are bicyclic nitroimidazoles (Singh et al., 2008). They were originally investigated as radiosensitizers for use in cancer chemotherapy (Agrawal et al., 1979), but the lead compound, PA-824, was also found to show potent bactericidal activity against multidrug resistant *M. tuberculosis* (Walsh et al., 1987; Nagarajan et al., 1989; Ashtekar et al., 1993) and now its congener delamanid is in phase III of clinical trials for the treatment of multidrug resistant tuberculosis. Although delamanid seems to be more active than PA-824 (Matsumoto et al., 2006), PA-824 has been widely used to describe the mechanism of action of this class of compounds as it is active against the *M. tuberculosis* complex (except *M. canettii*, Feuerriegel et al., 2011) not only toward the actively replicating but also against the nonreplicating bacteria. They inhibit the synthesis of mycolic acids and induce respiratory poisoning (Stover et al., 2000; Singh et al., 2008) through a peculiar mechanism of activation by the deazaflavin (cofactor F420) dependent nitroreductase (Ddn or Rv3547), that converts PA-824 into three primary metabolites (Manjunatha et al., 2009); the principal one is des-nitroimidazole (des-nitro), that generates reactive nitrogen species, including nitric oxide. Respiratory poisoning through nitric oxide release seems to be the main mechanism through which PA-824 exerts its anaerobic activity. Like cyanide, PA-824 dramatically shifted the predominant isoprenoid quinol/quinone ratio (MK9H2/MK9) in a time and concentration dependent manner. The effect of PA-824 on the respiratory complex under hypoxic nonreplicating conditions was also manifested by a rapid drop in intracellular ATP levels, therefore it has been hypothesized that upon its release within mycobacterial cells, toxic nitric oxide possibly reacts with cytochromes/cytochrome oxidase to interfere with the electron flow and ATP homeostasis under nonreplicating conditions (Manjunatha et al., 2009). No cross-resistance with current antituberculosis drugs has been observed, but mutations in any of the mycobacterial genes codifying the synthesis of cofactor F420 (*fgd1, fbiA, fbiB*, and *fbiC*) lead to resistance to PA-824 (Stover et al., 2000; Choi et al., 2001, 2002; Manjunatha et al., 2009). Also mutations in the *Rv3547* gene, encoding the Ddn, have been described in PA-824 resistant strains (Matsumoto et al., 2006; Manjunatha et al., 2009). Rv3547 is a protein with 151 amino acid residues with no detectable sequence homology with any other protein of known function and was shown to be nonessential (Sassetti et al., 2001). Complementation of the mutants with an intact *Rv3547* fully restored the ability of the mutants to metabolize PA-824. Q-203 (**Figure 7**) is grouped with delamanid and PA-824 for its structural similarity. Developed by Qurient Co., Q-203 is the first-in-class of imidazopyridine amide derivatives and it is current in phase Ib clinical trials (http://infectex.ru/en/products/q-203/). Q-203 was discovered after medicinal chemistry optimization and a high-throughput screening performed against infected human macrophages (Pethe et al., 2013). It targets the cytochrome *b* subunit (QcrB) of the cytochrome *bc*1 complex (complex III) which is an essential component of the *M. tuberculosis* respiratory electron transport chain, forcing *M. tuberculosis* to use the cytochrome *bd*, a terminal oxidase energetically less efficient (Lamprecht et al., 2016). Q-203 causes a rapid depletion of the intracellular ATP levels at 1.1 nM and is able to interfere with ATP homeostasis in nonreplicating *M. tuberculosis* at concentrations of < 10 nM, suggesting the inhibition of cytochrome *bc1* activity as its primary mode of action (Pethe et al., 2013). Recently, Jang et al. showed the involvement of efflux pumps in the off-target-based mechanism of resistance to Q-203 (Jang et al., 2017). Q-203 shows low frequency of drug resistance of *M. tuberculosis* and a favorable pharmacokinetic profile, which is important during long-term treatment (Pethe et al., 2013). Finally, TB-47 (**Figure 9**), developed by the Guangzhou Institutes of Biomedicine and Health, is structurally similar to Q-203 with the only difference represented by the substitution of the imidazopyridine ring with a substituted pyrazolopyrimidine, and is currently in the hit-to-lead optimization process. It has an interesting MIC against susceptible *M. tuberculosis* H37Rv of 0.003 μg/mL, whereas against 6 clinical drug-resistant isolates from China MICs were 0.06–0.12 μg/mL. In addition, it showed very good synergetic bactericidal effect with rifampicin and pyrazinamide. Although in the early development, it has been introduced here since its structural analogies, that will be commented below, with the above described antituberculosis drugs.

As in the case of bedaquiline, the structure of the molecules above described stimulates the discussion on how antituberculosis medicinal chemistry challenges the drug-likeness dogma. Delamanid consists of a nitroimidazooxazole attached to a lateral chain made of aromatic and aliphatic rings connected by a heteroatom such as oxygen, giving a stretched shape to the overall structure. Some of these chemical features are shared by PA-824 (the nitroimidazole core), Q-203 (lateral chain), and TB-47 (lateral chain). Delamanid presents only one violation of the Lipinski's “rule of five,” as its MW is >500 g/mol, with a borderline ClogP of 4.84. In general, high MW is connected to scarce solubility, high lipophilicity, and high metabolism rate, since the higher number of atoms increases the surface of metabolism. As in the case of bedaquiline, it can be rationalized that high molecular weight and high lipophilicity are necessary to obtain good activity toward mycobacteria. However, the characteristic that mainly stands against the drug-likeness of the molecule is the presence of the nitro group attached to the imidazooxazole ring, as in the case of PA-824 (Barry et al., 2004; Manjunatha et al., 2009), where the nitro moiety is attached to a imidazooxazine heterocycle. The nitro moiety is a standard red flag in medicinal chemistry campaigns, because of the intrinsic toxicity of this group (Kalgutkar et al., 2005; Boelsterli et al., 2006; Erve, 2006). Usually, in large chemical screenings, compound bearing this moiety would not pass *in silico* filters, and their advancement in the following steps of biological evaluation would be likely hampered.

**Figure 5:**
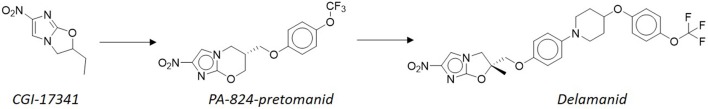
Evolution of the nitroimidazoles as anti-tuberculosis compounds.

Nitroaromatics are reduced to form reactive nitro, nitroso, nitroxyl radical, and aromatic N-oxide. These can induce oxidative stress, and lead to cellular death or induce mechanisms of inflammation. Although this mechanism of toxicity is quite well known, there are a number of drugs embodying a nitro group, that are currently marketed. Various 5- and 2-nitroimidazoles and 5-nitrofurans such as metronidazole, tinidazole, and nimorazole are known to be effective against a variety of protozoan and bacterial infections in humans and animals (Raether and Hänel, 2003). These compounds, however, are also known to possess mutagenicity, because the metabolized nitro group at the level of DNA may cause DNA viscosity reduction as well as DNA damage, representing a constant warning toward their watchful use (Rodriguez et al., 2002). This issue was taken into careful consideration during the early drug discovery stage of delamanid development. CGI-17341 (Figure 5), the precursor of the nitroimidazoles, was found to show mutagenic properties (Nagarajan et al., 1989; Ashtekar et al., 1993). Therefore, the medicinal chemistry campaign was not focused on improving the antituberculosis activity, but rather, on decreasing the rate of mutagenicity, that was constantly monitored through a bacterial reverse mutation test (Matsumoto et al., 2007). After many rounds of modifications, it was possible to decrease the mutagenicity rate after introducing heteroatoms instead of alkyl chain at the C-2 position. Among the non-mutagenic derivatives, PA-824 was also found to maintain potent antituberculosis activity, and the following hit-to-lead optimization led to the discovery of delamanid (Sasaki et al., 2006). This successful example, however, must be considered as an exception in the landscape of drug discovery, as the pursuing of a structure-toxicity relationship, rather than structure-activity relationship (SAR), most of the time does not lead to similar satisfactory results. Although the presence of somebody standing out of the crowd (Boechat et al., 2015), the extent to which the presence of a nitro group can be tolerated in a molecule that is supposed to be administered for several months, remains a matter of debate and the use of nitroaromatic drugs is generally not recommended for a long-term treatment (Patterson and Wyllie, 2014). In spite of that, besides delamanid and PA-824, there is a quite consistent number of molecules in the tuberculosis drug pipeline containing a nitro group, such as nitazoxanide (Shigyo et al., 2013) currently in phase II clinical trials, mentioned for the sake of information, and the recently released benzothiazinones BTZ-043 and PBTZ-169, that will be discussed later more in details. The second structural peculiarity of delamanid, shared with PA-824 and to a larger extent with Q-203 and TB-47, is the presence of an extended chain of aromatic and aliphatic rings characterized by a *p*-trifluromethoxy phenyl substitution. Rather surprisingly, the *p*-trifluoromethoxy moiety can be found only in 3 marketed drugs (source: https://www.drugbank.ca/), but, on the other hand, it is found in 5 molecules in the tuberculosis pipeline. If the trifluoromethyl group, a chemical congener of the trifluoromethoxy, is considered in the count, the number raises to 6. This moiety is seldom used in medicinal chemistry and the reason of its scarce use is generally attributed to its ability to greatly increase lipophilicity. Again, the use of groups with lipophilicity-enhancer characteristics such as the trifluormethoxy, thrifluoromethyl, or bromine in the case of bedaquiline, although not common in other therapeutic backgrounds, becomes the standard in antituberculosis research. Finally, it must be also considered that, differently from delamanid, Q-203 (ClogP = 7.10 and MW > 500 g/mol) and TB-47 (ClogP 6.61 and MW = 538.56 g/mol), present two violations of Lipinski's “rule of five”.

#### SQ-109, AU-1235, NTDI-304, and NTDI-349

**Figure 6:**
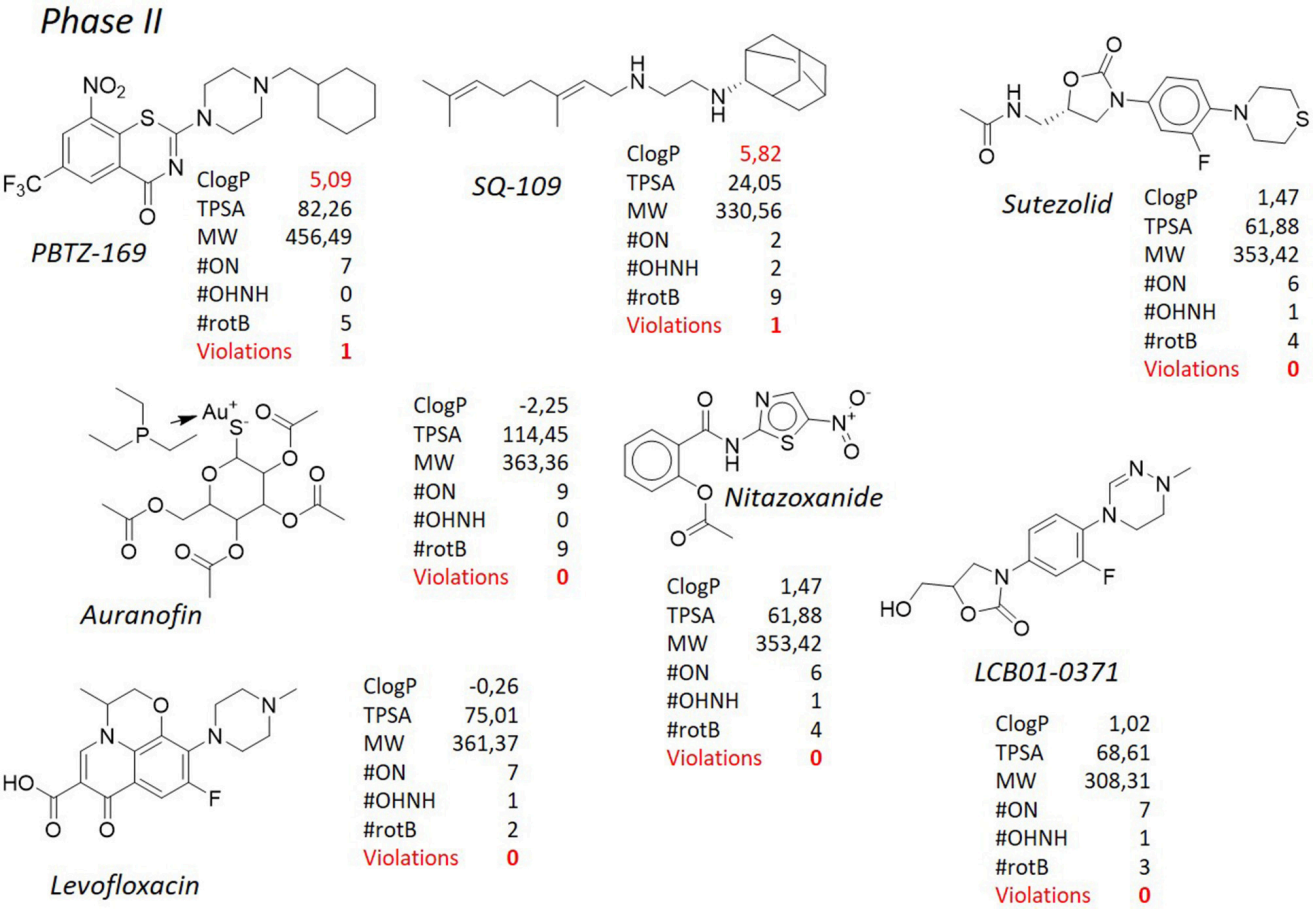
TB pipeline: molecules in phase II clinical trials. Source: https://www.newtbdrugs.org/pipeline/clinical. For each molecule physicochemical characteristics are reported, along with the violation to the “rule of five.” Pysicochemical properties calculated at http://www.molinspiration.com/cgi-bin/properties.

SQ-109 (Figure 6), developed by Sequella, Inc., is a 1,2-ethylene diamine compound roughly similar to ethambutol, a first-line antibacterial used in the treatment of tuberculosis, and is currently in Phase IIb-III clinical trials (http://infectex.ru/en/products/sq-109/). SQ-109 was discovered using two high-throughput screening assays, initially by MIC determination and later using an *iniBAC* promoter based cell wall inhibition bioluminescence assay (Protopopova et al., 2005; Sacksteder et al., 2012). SQ-109 has been reported to act by inhibiting the mycobacterial trehalose monomycolate transporter MmpL3, involved in cell wall biosynthesis (Tahlan et al., 2012). However, it is also active against fungi and bacteria that are devoid of mycolic acids (Onajole et al., 2011; Sacksteder et al., 2012) and shows activity against non-replicating cells, that, by definition, shut down the synthesis of the outer membrane (Li K. et al., 2014). This suggests a pleomorphic mode of action, and indeed further investigation on SQ-109 led to the discovery that SQ-109 has 3 unique mechanisms of action (Sacksteder et al., 2012) and identified additional inhibition of menaquinone synthesis (MenA and MenG), and inhibition of cellular respiration and ATP synthesis, in part due to dissipation of the PMF (Li K. et al., 2014; Li W. et al., 2014; Li et al., 2017). Moreover, it acts synergistically *in vitro* and in an animal model when combined with rifampicin, isoniazid, or bedaquiline (Chen et al., 2006; Nikonenko et al., 2007; Reddy et al., 2010; Sacksteder et al., 2012) and in pulmonary tuberculosis patients in combination with rifampicin (Heinrich et al., 2015). SQ-109 presents some pharmacological limitations due to its amphipathic structure, nevertheless, the results of preclinical studies and a series of early phase clinical studies have showed that SQ-109 has a good safety and tolerability, which is important in the case of long-term combined treatment. From the structural point of view, SQ-109 is simplified by the 1,2-ethylene diamine structure, as it was developed in order to improve the activity of ethambutol (Lee et al., 2003; Bogatcheva et al., 2006). During the design phases, it was noticed that the majority of active compounds were considerably lipophilic, and for instance the final ClogP for SQ-109 is 5.82, representing the only violation of the Lipinski's “rule of five.” The most frequently occurring fragments in the active compounds were: a highly α-branched aliphatic moieties, 2,2-diphenylethyl and 3,3-diphenylpropyl fragments, tricyclic skeletons derived from adamantane-containing amine monomers, myrtanylamine, isopinocamphylamine and isoprenoid structures. This SAR led to the design and synthesis of a new >30,000 diamine library of molecules, that, on turn, led to the discovery of novel scaffolds with activity against *M. tuberculosis*. Although the presence of two hydrophilic amino groups, the pattern of substituents was arranged in order to maintain the adamantyl substituent or the geranyl one. Aromatic substituents, with a considerable drop in lipophilicity, are detrimental for the activity (Protopopova et al., 2005). Of particular note, as mentioned, it is the use in SQ-109 of the adamantyl substituent attached to one of the amino groups. Adamantane is a bulky and highly hydrophobic structure, that confers not only a considerable lipophilicity, but also a certain degree of steric hindrance. Due to these characteristics, adamantane is uncommon in the drugs scenario, and only 6 drugs reporting this moiety (adapalene, rimantadine, amantadine, memantine, vildagliptin, saxagliptin) are currently marketed (source: https://www.drugbank.ca/). Those compounds used as antivirals (rimantadine, amantadine, memantine) are very similar and share no significant diversity, whereas in vildagliptin and saxagliptin the adamantane ring is used for its steric hindrance rather than for its enhancement of lipophilicity. In spite of that, adamantane is considered in medicinal chemistry a “lipophilic bullet,” and as such it has been used according to an “add-on” strategy to known pharmaceuticals or as a replacement for other lipophilic groups (Wanka et al., 2013). This group has been added, among the others, to antidiabetic drugs (Gerzon et al., 1963), anabolic steroids (Rapala et al., 1965), antimalarials (Gerzon and Kau, 1967), and also penicillins (Kovtun and Plakhotnik, 1987). None of these experimental derivatives has reached the clinic, however in many cases a considerable improvement of the activity was obtained. Therefore, it is not surprising that adamantane was exploited to prepare improved antituberculosis analogs. Indeed, along with SQ-109, also AU-1235 (Grzegorzewicz et al., 2012) an urea derivative, is currently in the hit-to-lead optimization process for the treatment of tuberculosis (**Figure 9**). Another class of antituberculosis molecules for which the “add-on” strategy of a lipophilic bullet such as the adamantane ring has led to improved activity is that of indole-2-carboxamides. NITD-304 (ClogP 4.94) and NITD-349 (ClogP 3.91) are two antitubercular candidates developed by Novartis for which the hit-to-lead optimization is currently ongoing (**Figure 9**, Rao et al., 2013; Harrison, 2014). Although these two derivatives are devoid of the adamantyl substituent, in the first disclosure of the indole-2-carboxamides as potent antituberculosis agents (Lun et al., 2013; Onajole et al., 2013) the SAR clearly highlighted that those compounds bearing an adamantyl, an octyl, or an eptyl ring were the most active of the series, whereas when the lipophilic nature of the ring was disrupted by a nitrogen atom, the activity was lost (**Figure 9**). Even odder is the fact that all of these molecules (SQ-109, AU-1235, and indolcarboxamides) are inhibitors of the mycolic acid transporter MmpL3. The mycobacterial membrane protein Large (MmpL) family is involved in transportation of metabolites from the cytosol of *M. tuberculosis* and plays an important role in its survival and pathogenesis (Domenech et al., 2005). The reason why so different structures share the same molecular target [of note, BM-212 (La Rosa et al., 2012), a pyrrole derivative, is as well an MmpL3 inhibitor], is still a matter of debate (Li et al., 2017), however the only feature that they have in common is the high lipophilicity, and that is understandable since the role of MmpL3 in exporting mycolic acids.

#### BTZ-043 and PBTZ-169

The benzothiazinones are a new class of antituberculosis drug candidates that inhibit the decaprenylphosphoryl-β-d-ribose 2′-epimerase (DprE1), an essential enzyme involved in arabinan biosynthesis needed for the bacterial cell wall (Makarov et al., 2009). Further studies showed that BTZ-043 (**Figure 8**), the lead compound of this class and currently in phase II of clinical trials (http://panacea-tb.net/clinical-studies/panacea-studies/), is activated through the reduction of an essential nitro group to a nitroso derivative, which can react with a cysteine residue in DprE1 (Trefzer et al., 2010). Manina et al. (2010) have proposed an alternative mechanism of resistance using *M. smegmatis* in which the overexpression of the nitroreductase NfnB leads to the inactivation of BTZ-043 through the reduction of an essential nitro group to an amino group. Although *M. tuberculosis* apparently lacks nitroreductases able to reduce this drug, this finding can be useful for development of new benzothiazinone derivatives with improved activity (Manina et al., 2010). Along with MmpL3 previously described, also DprE1 is considered a promiscuous target, since the relatively high number of molecules, also belonging to different chemical series, that have shown to inhibit it (Lechartier et al., 2014; Chiarelli et al., 2016). PBTZ-169 (Figure 6), that shares the same mechanism of action of BTZ-043, is a piperazinobenzothiazinone structurally related to its parent compound, with improved pharmacokinetic characteristics and, especially, with better synthetic accessibility since the lack of stereocenters. Besides being very selective toward mycobacterial species, PBTZ-169 and BTZ-043 are extremely active alone (low nanomolar/subnanomolar range) and, also, PBTZ-169 has shown additive or synergistic effects with many tuberculosis therapeutic agents, both marketed or in development (Makarov et al., 2014). Both compounds have lipophilic nature, with PBTZ-169 showing a violation of the Lipinski's “rule of five” (ClogP = 5.06), although not as striking as in the cases of bedaquiline and delamanid. As mentioned before, the nitro functional group is not amendable for this class of compounds. It is selectively reduced to a nitroso group, which then reacts with a key active site cysteine residue of DprE1 (Cys387 in *M. tuberculosis*) to form a semi-mercaptal adduct that inhibits the enzyme (Trefzer et al., 2010) and a mutation to the Cys387 greatly reduces the antituberculosis activity of benzothiazones. Although the nitro group might decrease the overall drug likeness of the molecule, this reductive pathway seems to be absent in human metabolism, resulting in negative Ames test for DNA mutagenesis and anticipating the lack of toxicity in mammals. Despite these considerations, there have been several attempts to substitute the nitro group (Makarov et al., 2015). In particular, some 8-pyrrole-benzothiazinones retained significant antituberculosis activity, with MICs of 0.16 μg/mL against *M. tuberculosis*, but unfortunately not showing efficacy in a mouse model of acute infection. Likely, the lack of the irreversible inhibition of the target enzyme is crucial in order to reach the desired activity. Also in this case, the substitution of the nitro group with a heterocycle such as a pyrrole leads to a substantial increase of the lipophilicity of the molecule (ClogP PBTZ-169 = 5.09 vs. ClogP PyrBTZ-02 = 5.81), corroborating the correlation between lipophilicity and activity. Further analyzing the SAR of benzothiazinones, it appears evident that a meta electron–withdrawing group, coupled to the nitro-aromatic feature, is necessary to obtain the required potency. Again, among the many compounds tested, those substituted with lipophilic EWGs (CF_3_, ClogP = 5.09 and Cl, ClogP = 4.87) are more active than those where a CN (ClogP = 3.95) and an H (ClogP = 4.24) are present.

**Figure 7:**
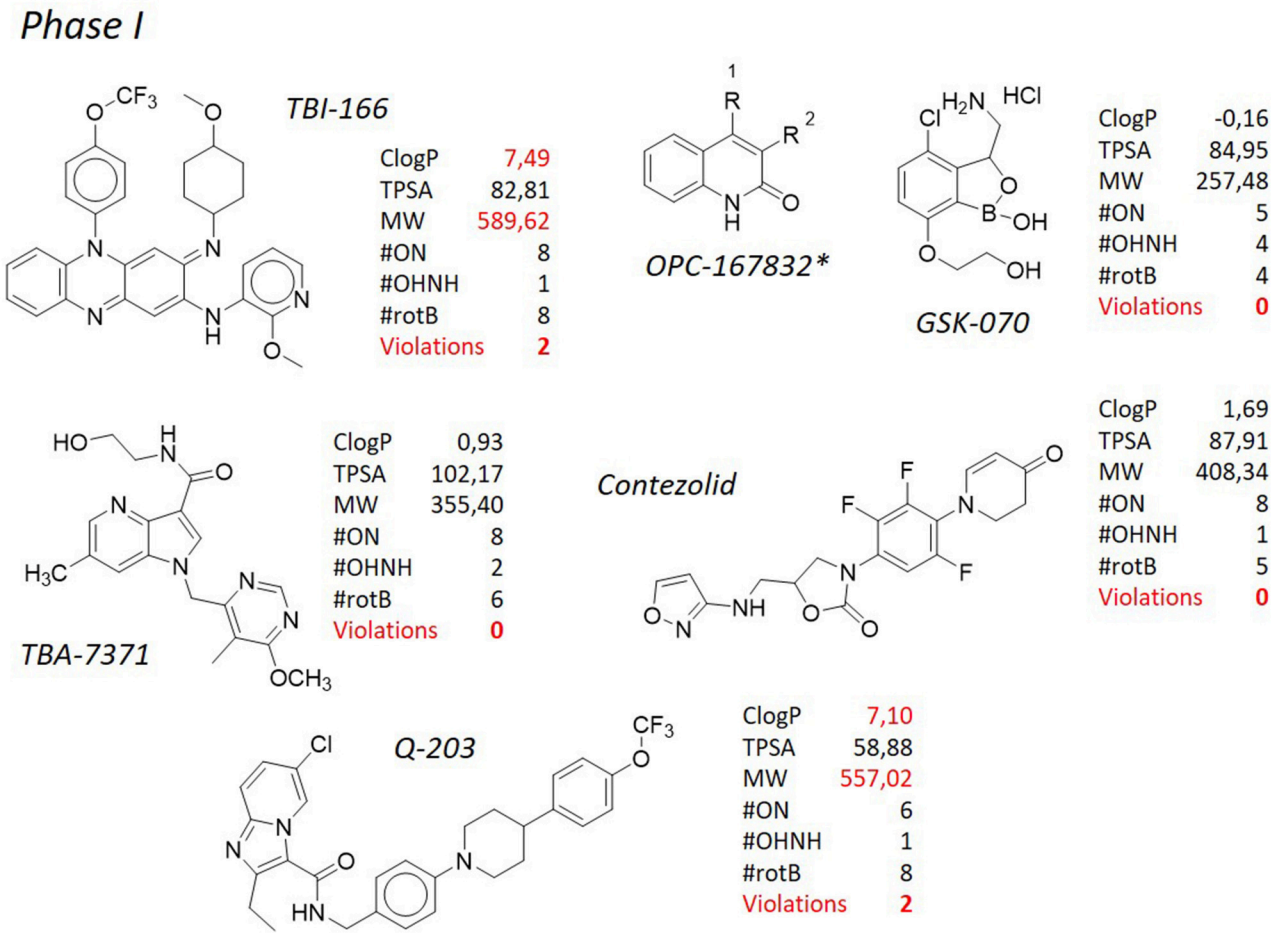
TB pipeline: molecules in phase I clinical trials. Source: https://www.newtbdrugs.org/pipeline/clinical. For each molecule physicochemical characteristics are reported, along with the violation to the “rule of five.” Physicochemical properties calculated at http://www.molinspiration.com/cgi-bin/properties. *Structure not released.

**Figure 8:**
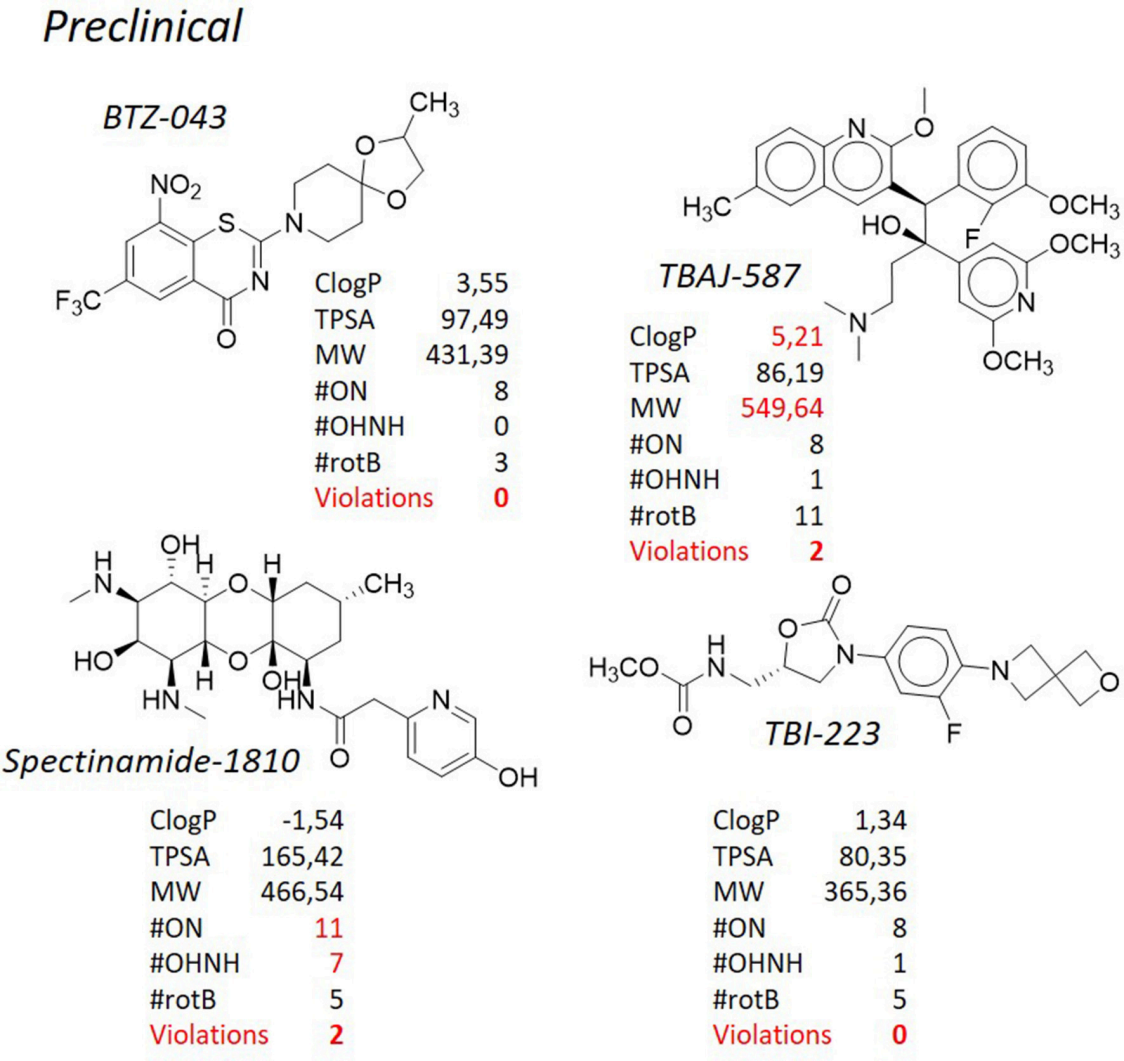
TB pipeline: some molecules in pre-clinical phases. Source: https://www.newtbdrugs.org/pipeline/clinical. For each molecule physicochemical characteristics are reported, along with the violation to the “rule of five.” physicochemical properties calculated at http://www.molinspiration.com/cgi-bin/properties.

**Figure 9:**
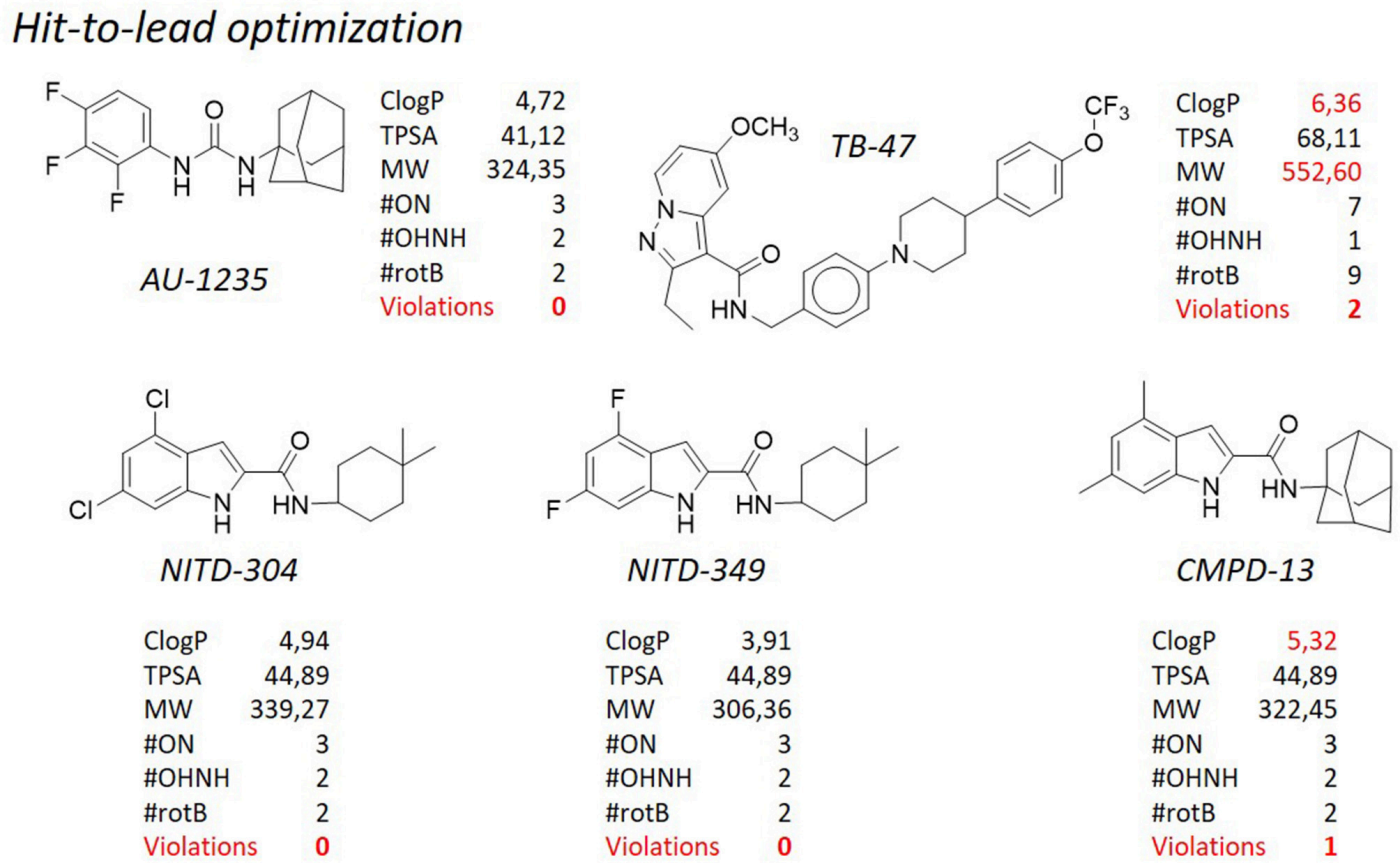
TB pipeline: some molecules in the hit-to-lead optimization phase. Source: https://www.newtbdrugs.org/pipeline/clinical. For each molecule physicochemical characteristics are reported, along with the violation to the “rule of five.” Physicochemical properties calculated at http://www.molinspiration.com/cgi-bin/properties.

#### Clofazimine and TBI-166

Clofazimine (Figure 4), belonging to the riminophenazine antibiotic class, is a greasy dye introduced in the 1960 for the treatment of leprosy along with dapsone. The structure of clofazimine consists of a phenazine scaffold suitably substituted at the nitrogen and at one of the phenyl ring with aromatic substituents. This confers high lipophilic character to the molecule (ClogP = 8.43), therefore scientists have reconsidered its use toward *M. tuberculosis*. Its antituberculosis activity has been recently demonstrated (Gopal et al., 2013), either alone or in combination with other antituberculosis compounds (Diacon et al., 2015; Tang et al., 2015; Yang et al., 2017). Because of these findings, clofazimine is currently being studied in phase III clinical trials as a component for new regimens to treat multidrug resistant tuberculosis, in combination with other marketed or developing molecules; on the other side, novel analogs able to improve the physicochemical profile of the parent compound are under investigation. In particular, several side effects, such as discoloration of the skin, can be attributed to the extremely high lipophilicity of the molecule, therefore a series of clofazimine analogs bearing a C-2 pyridyl substituent were designed and synthesized with the goal of improving the safety profile by lowering the lipophilicity. One of those, TBI-166 (Figure 7), developed by TB Alliance in partnership with the Institute of Materia Medica (IMM) in Beijing (Zhang et al., 2012, 2014), represents the only case in which an attempt of improving of an antituberculosis agent was pursued through lowering the ClogP. TBI-166, currently a preclinical development candidate, has demonstrated potent activity against *M. tuberculosis* (MIC = 0.016 μg/mL) *in vitro* and an activity comparable to that of clofazimine in an multidrug resistant tuberculosis experimental mouse infection model, although with significantly reduced skin discoloration potential. Although reduced, lipophilicity is still way above the prudential value claimed by the Lipinski's “rule of five” (ClogP = 7.49), and it is coupled to another violation consisting in the high MW (589.62). Also in this case, it is worth of mention the presence of the *p*-trifluoromethoxy group attached to the phenazine nitrogen, a functional group already discussed above, that, to some extent, could be considered a “privileged moiety” in the design and synthesis of antituberculosis compounds.

#### Thioridazine, verapamil, and UPAR-174

**Figure 10:**
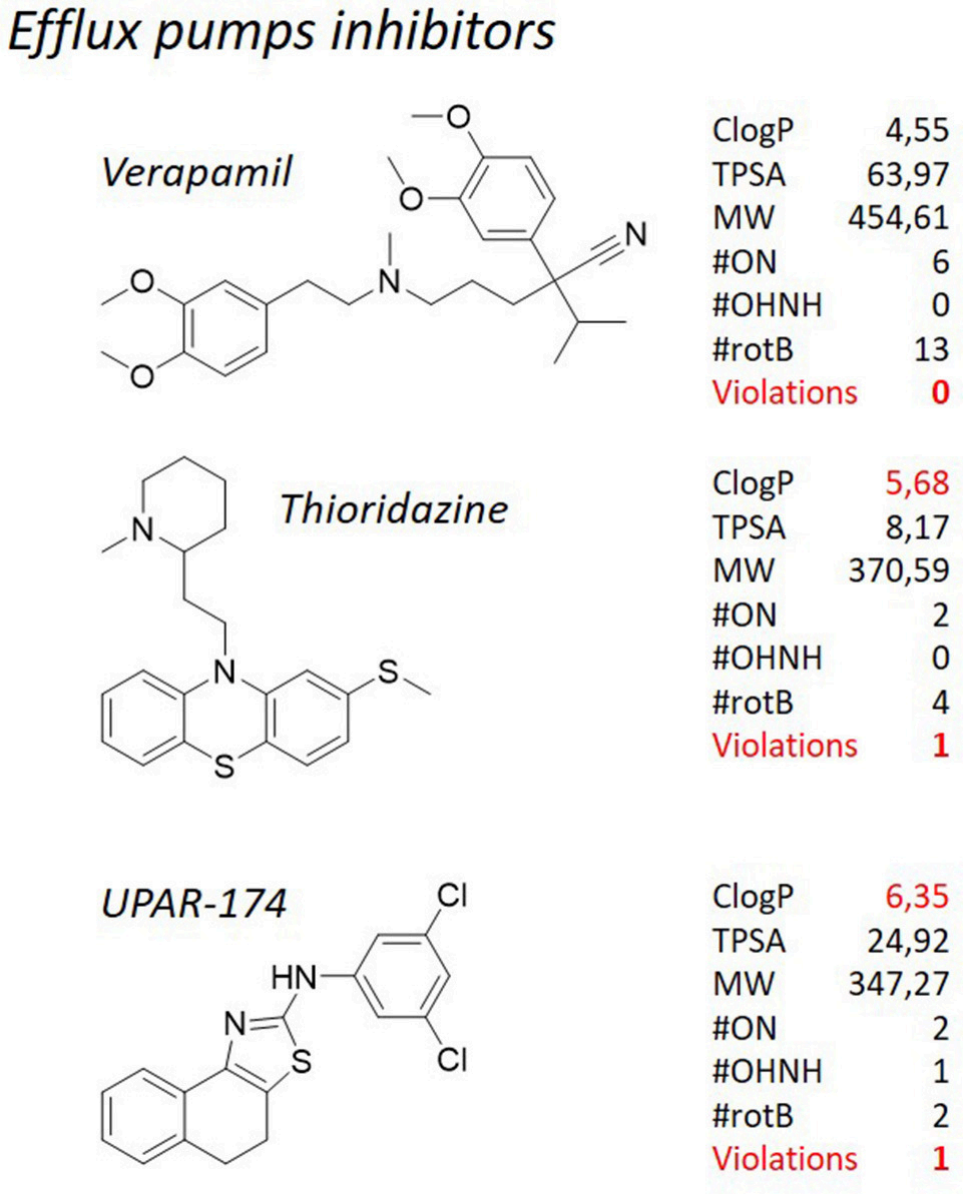
TB pipeline: efflux pumps inhibitors for *M. tuberculosis*. For each molecule physicochemical characteristics are reported, along with the violation to the “rule of five.” Physicochemical properties calculated at http://www.molinspiration.com/cgi-bin/properties.

We have tried to demonstrate that adherence to the Lipinski's “rule of five” is hardly attainable for antibacterials in general, and for antituberculosis drugs in particular. This notion not only applies to those molecules designed to kill mycobacteria, but, to some extent, it can be extended also to some antituberculosis adjuvant therapies. We have already discussed herein the use efflux pump inhibitors (Van Bambeke et al., 2006; Pule et al., 2016) as an adjuvant strategy for the treatment of tuberculosis. Our attention was focused on verapamil, an antiarrhythmic drug, and thioridazine (**Figure 10**), a neuroleptic with efflux inhibitory properties (Martins et al., 2007; Rodrigues et al., 2008) that in a clinical trial in Argentina was used for the treatment of patients with XDR-TB in combination with antituberculosis drugs under compassionate bases, with encouraging results (Abbate et al., 2012). Even though these compounds are not supposed to actually kill the cells, nevertheless they have to interact with the greasy cell wall. Therefore, it is not surprising that thioridazine, that is currently one of the most studied mycobacterial efflux pumps inhibitors, has a high ClogP of 5.68. From the structural point of view, it consists of a phenothiazine substituted with a thioether group at the C-2, and connected by the nitrogen atom to a *N*-methylpiperidine through an ethylene linker. The polycyclic ring structure, which is important for the neurological action of the class, contributes to the lipophilicity of the compound and can be substituted with various groups on C-2. Chlorpromazine, another neuroleptic with efflux inhibitory properties, was tested along with its metabolites against *M. smegmatis* and exhibited synergistic activity when administered in combination with known antituberculosis drugs (Kigondu et al., 2014a,b). Also chlorpromazine, that is structurally related to thioridazine, shows a violation of the Lipinsky's “rule of five” with its ClogP (5.03). The amphiphilic nature of these compounds is important for their calmodulin inhibitory activity but may also play a role in their interaction with efflux pumps. The lipophilic cationic nature of the molecule determines the degree of interaction with lipid membranes and thus influences the distribution patterns of the class (Kapp et al., 2018). Verapamil is known as the most potent inhibitor of efflux systems in *M. tuberculosis*, but its use as co-adjuvant in therapy is not recommended due to its mechanism of therapeutic action, that is the block of the calcium channel. Verapamil, marketed as a racemic mixture, is made of a tertiary amine substituted with a methyl group and two substituted aromatic rings connected through an aliphatic spacer. Differently from the many molecules described here, this molecule does not violate any of the Lipinski's “rule of five” (ClogP = 4.55 and MW = 454.61 Da), although these values are fairly above the average. Recently, we have reported a medicinal chemistry campaign aiming at lowering the toxicity of thioridazine and, at the same time, improving its efflux inhibitory properties and drug-likeness, through a ligand-based drug design approach (Pieroni et al., 2015a; Costa et al., 2016). Despite the good preliminary results, it could be noticed that a drop in the lipophilicity of the synthesized derivatives has detrimental effects on the overall performance of the molecules. Keeping this notion under consideration, other efforts were dedicated to the disclosure of novel chemical structures as efflux pump inhibitors. During a screening campaign of a series of 2-aminothiazoles, that led to potent antituberculosis chemotypes (Pieroni et al., 2014, 2015b; Azzali et al., 2017), UPAR-174 (**Figure 10**), a tricyclic structure embodying a scaffold, was found to show potent efflux inhibitory properties toward *M. tuberculosis*, in the same range of verapamil, and better than thioridazine (Machado et al., unpublished). Also in this case, the high lipophilicity of the molecule (ClogP = 6.60) was found to be a key parameter in order to guarantee high activity at the site of infection. Indeed, it was found to be able to penetrate the macrophage cell wall, and to facilitate killing of *M. tuberculosis* inside the macrophage in synergy with rifampicin and isoniazid administered at sub-lethal concentrations.

## Concluding remarks

The search for novel compounds to be used either alone or in combination with other antibiotics to treat tuberculosis and drug resistant tuberculosis infections has become a major goal of drug discovery programs. In this review, we have covered the recent advances and challenges in tuberculosis drug discovery and provide an overview on how *M. tuberculosis* shifts the dogmatic drug-likeness dictated by the Lipinski's “rule of five” to an “activity-through-lipophilicity” vision of medicinal chemistry. It seems that, either for the direct treatment or to pursue an adjuvant strategy, high lipophilicity is a key parameter that must be taken into consideration when medicinal chemistry efforts are made. Out of the 26 molecules representing the TB pipeline in early discovery stages, pre-clinical and clinical studies (Figures 4, 6–9), 11 show at least one violation of the Lipinski's “rule of five”, and the majority of them have functional groups in their structure that are seldom used because counter-productive in medicinal chemistry settlements. Those molecules that are exempt from violations, either they are repurposed drugs (levofloxacin, rifampicin at higher doses) or they are novel chemical entities but deriving from known and validated antibacterial chemical classes (such as oxazolidindiones and quinolones), resulting in scarce novelty. Nevertheless, especially for quinolones, evidences are that a lipophilic enrichment of known structures is likely to improve the potency toward *M. tuberculosis* (Tabarrini et al., 2012; Fan et al., 2018). By virtue of their high lipophilicity, that allows for strong drug-membrane interactions, some of the new molecules in the pipeline act through the inhibition of membrane proteins in addition to collapsing the PMF, and are highly potent against *M. tuberculosis*, both actively replicating and dormant. The inhibition of energy metabolism using combination of drugs targeting components or pathways such as the PMF in *M. tuberculosis* presents several advantages as rapid bactericidal action, activity against replicating and non-replicating strains, and low propensity for the development of drug resistance. In addition, these combinations of membrane active agents may be highly synergistic with current first and second line drugs, allowing to reach the main aim of shortening tuberculosis treatment duration. Therefore, our opinion is that drug development efforts should also focus on those compounds to which resistance could not be selected, along with the optimization and development of hits to which resistance can reasonably occur. Perhaps, bedaquiline is the most significant example of what this manuscript wants to deliver: adherence to the “rule of five” may or may have resulted in the loss of opportunities, in particularly for a difficult pathogen as *M. tuberculosis*. Bedaquiline is the first approved drug for the treatment of tuberculosis after decades of stagnation, but it would have failed to pass any *in silico* filter that is commonly used to skim *ex novo* synthetic chemical libraries for further advancement and/or the eye inspection of a skilled medicinal chemist.

## Author contributions

All authors collated data and prepared the manuscript. DM and MV initially formulated the concept of the review. MG contributed to the writing. MP coordinated the work and collected the data.

### Conflict of interest statement

The authors declare that the research was conducted in the absence of any commercial or financial relationships that could be construed as a potential conflict of interest.
